# "A cross-sectional analysis of four common clinical decision rules for pulmonary embolism": The need for integrated and evolving approaches

**DOI:** 10.34172/jcvtr.025.33664

**Published:** 2025-12-17

**Authors:** Erfan Banisefid, Elnaz Javanshir

**Affiliations:** ^1^Student Research Committee, Tabriz University of Medical Sciences, Tabriz, Iran; ^2^Cardiovascular Research Center, Tabriz University of Medical Sciences, Tabriz, Iran

## Dear Editor,

 I read with interest the article by Hassani et al titled “A cross-sectional analysis of four common clinical decision rules for pulmonary embolism, Mashhad, Iran”,^1^ evaluating the diagnostic value of four widely used clinical decision rules (CDRs). The authors’ attempt identifying a screening pathway is valuable. However, there are some concerns regarding the validity of this research.

 First is the exclusion of serum D-dimer, which is an important part of current evidence-based pulmonary embolism (PE) workups.^2,3^ CRDs such as the Wells and Geneva scores are no longer considered to be functional in isolation; these scores are globally preferred to be coupled with D-dimer testing to safely rule out PE in low-risk patients.^4^ Although laboratory factors aren’t used in CDRs for faster evaluation, neglecting this makes the findings less applicable and raises the risk of overestimating CT angiography utilization. Al Dandan et al’s study found that inconsistent D-dimer documentation can impact diagnostic quality.^5^ Hassani et al^1^ could emphasize more on this important gap, especially since similar challenges may exist across the region.

 Moreover, the study’s suggestion to utilize the Wells score at first, and then the revised Geneva score as a subsequent filter, could be presenting an unverified diagnostic approach. These CDRs are grounded in distinct theoretical foundations and are not intended to be combined. This two-step method is lacking verification and could complicate clinical approach instead of simplifying it. Conversely, newer methods like the YEARS algorithm, studied by van Maanen et al present a more effective and straightforward model to rule out PE.^6^ The YEARS by integrating three clinical factors with age-adjusted D-dimer thresholds, was shown to effectively decrease unnecessary imaging referrals by almost 50%.

 Also, the limited sample size, unclear inclusion criteria, and the unavailability of confidence intervals for sensitivity and specificity estimates are restricting the statistical strength of the results. Relying on accuracy as the main performance metric can be misleading, as it fails to consider the clinical implications of false positives or false negatives. Other metrics, for example likelihood ratios or the area beneath the ROC curve could enhance the diagnostic evaluation. A paragraph about practical implementation difficulties, such as clinician knowledge of scoring systems, incorporation into electronic medical records, and time limitations in emergency care is also absent. These elements are mentioned as obstacles in the literature, notably by Al Dandan et al.^5^

 Although the article provides valuable region-specific data, it lacks parttaking with the new diagnostic approaches for PE. Future studies should concentrate on integrating CDRs with adaptable biomarker thresholds, assessing tools such as YEARS or PEGeD, and evaluating effectiveness in various environments including outpatient clinics and primary care.

## Competing Interests

 The author declares no conflict of interest in this study.

## Ethical Approval

 Not applicable.
